# Supplementary material for: Inhaled corticosteroids do not adversely impact outcomes in COVID-19 positive patients with COPD: An analysis of Cleveland Clinic’s COVID-19 registry
Source: PLoS One. 2021 Jun 3;16(6):e0252576. doi: 10.1371/journal.pone.0252576 (PMC8174679; doi:10.1371/journal.pone.0252576)
Supplement: S1 Table — (DOCX) [file pone.0252576.s001.docx]

**S1 Table. ICD9 and 10 codes used for diagnosis of medical conditions or outcomes.**

| **Diseases** | **ICD9** | **ICD10** |
| --- | --- | --- |
| Asthma | 493.00 493.01 493.02 493.11 493.12 493.20 493.21 493.22 493.81 493.82 493.90 493.91 493.92 | J45 J45.20 J45.21 J45.22 J45.30 J45.31 J45.32 J45.40 J45.41 J45.42 J45.50 J45.51 J45.52 J45.901 J45.902 J45.909 J45.990 J45.991 J45.998 |
| COPD | 491 | J42 J41.0 J41.1 J41.8 J44.9 J44.1 J44.0 J41.8 J42 J43.0 J43.1 J43.2 J43.9 J43.8 |
| Congestive heart failure | 39891 4280 4281 42820 42821 42822 42823 42830 42831 42832 42833 42840 42841 42842 42843 4289 | I0981 I130 I132 I110 I501 I5020 I5021 I5022 I5023 I5030 I5031 I5032 I5033 I5040 I5041 I5042 I5043 I509 |
| Hypertension | 4011 4019 40200 40201 40210 40211 40290 40291 4030 40300 40301 4031 40310 40311 4039 40390 40391 4040 40400 40401 40402 40403 4041 40410 40411 40412 40413 4049 40490 40491 40492 40493 4010 40501 40509 40511 40519 40591 40599 4372 | I110 I119 I152 I158 I159 E000 E001 E002 E009 E018 E02 E030 E031 E032 E033 E038 E039 |
| Diabetes mellitus | 24900 25000 25001 7902 79021 79022 79029 7915 7916 V4585 V5391 V6546 24901 24910 24911 25002 25003 25010 25011 25012 25013 24940 24941 25040 25041 25042 25043 24950 24951 25050 25051 25052 25053 24960 24961 25060 25061 25062 25063 24970 24971 25070 25071 25072 25073 24990 24991 25090 25091 24920 24921 24930 24931 24980 24981 25020 25021 25022 25023 25030 25031 25032 25033 25080 25081 25082 25083 25092 25093 | E0800 E0801 E0810 E0811 E089 E0900 E0901 E0910 E0911 E099 E1010 E1011 E109 E1100 E1101 E1110 E1111 E119 E1300 E1301 E1310 E1311 E139 O24011 O24012 O24013 O24019 O2402 O2403 O24111 O24112 O24113 O24119 O2412 O2413 O24311 O24312 O24313 O24319 O2432 O2433 O24811 O24812 O24813 O24819 O2482 O2483 O24911 O24912 O24913 O24919 O2492 O2493 |
| Pulmonary embolism |  | I26 I26.99 I26.94 I26.93 I26.92 I26.90 I26.9 I26.09 I26.02 I26.01 I26.0 |
| Sepsis |  | A41** R65** |
| Pneumonia |  | J12** J13 J14 J15** J16** J17 J18** |
| Shock |  | R57** R65.21 T79.4 T78.2 T78.0 T80.5 T75.4 A48.3 T81.1 3E030XZ 3E040XZ |
| Acute kidney injury |  | N17** |
| Acute liver failure |  | K720* K712 |
| DIC and coagulopathy |  | D65 |
| Mechanical ventilation |  | 0BH17EZ 0BH18EZ 5A1935Z 5A0945Z 5A0955Z |
| Non invasive positive pressure ventilation |  | 5A09357 5A09457 5A09458 5A09358 5A09557 5A09558 |
