# Supplementary material for: Inhaled corticosteroids do not adversely impact outcomes in COVID-19 positive patients with COPD: An analysis of Cleveland Clinic’s COVID-19 registry
Source: PLoS One. 2021 Jun 3;16(6):e0252576. doi: 10.1371/journal.pone.0252576 (PMC8174679; doi:10.1371/journal.pone.0252576)
Supplement: S2 Table — (DOCX) [file pone.0252576.s002.docx]

**S2 Table. Clinical Characteristics of all patients with COPD (inpatient and outpatient) who tested positive for COVID-19 based on OCS usage.**

|  | No OCS | OCS | p |
| --- | --- | --- | --- |
| n | 300 | 988 |  |
| Demographics | | | |
| Male gender (%) | 133 (44.3) | 366 (37.1) | 0.029 |
| Race (%) |  |  | 0.209 |
| Black | 106 (35.7) | 349 (35.8) |  |
| Other | 12 (4.0) | 33 (3.4) |  |
| White | 163 (54.9) | 527 (54.1) |  |
| Hispanic | 16 (5.4) | 65 (6.7) |  |
| Age (mean (SD)) | 65.73 (16.61) | 63.11 (14.73) | 0.009 |
| BMI (mean (SD)) | 31.10 (8.98) | 32.23 (8.60) | 0.05 |
| Smoking status |  |  | <0.001 |
| Current | 32 (11.0) | 83 (8.5) |  |
| Former | 245 (84.5) | 881 (90.5) |  |
| Never | 2 (0.7) | 3 (0.3) |  |
| Medications (%) | | | |
| LAMA | 21 (7.0) | 169 (17.1) | <0.001 |
| LAMA/LABA | 5 (1.7) | 58 (5.9) | 0.005 |
| Inhaled corticosteroid | 65 (21.7) | 503 (50.9) | <0.001 |
| Comorbidities (%) | | | |
| Asthma | 39 (13.0) | 340 (34.4) | <0.001 |
| Congestive heart failure | 62 (20.7) | 266 (26.9) | 0.035 |
| Hypertension | 215 (71.7) | 756 (76.5) | 0.103 |
| Diabetes | 147 (49.0) | 576 (58.3) | 0.005 |
| Obesity | 142 (48.0) | 534 (54.2) | 0.071 |
| Outcomes (%) | | | |
| Admission after positive | 63 (21.0) | 308 (31.2) | 0.001 |
| Month of COVID positivity (%) |  |  | 0.091 |
| March | 15 (5.0) | 50 (5.1) |  |
| April | 54 (18.0) | 137 (14.0) |  |
| May | 50 (16.7) | 152 (15.6) |  |
| June | 46 (15.3) | 105 (10.8) |  |
| July | 90 (30.0) | 347 (35.6) |  |
| August | 45 (15.0) | 184 (18.9) |  |
| Data are presented as n (%) for categorical variables and mean [SD] for continuous variables. Month of COVID positivity represents the month during which the COVID test was positive. | | | |
