# Supplementary material for: Inhaled corticosteroids do not adversely impact outcomes in COVID-19 positive patients with COPD: An analysis of Cleveland Clinic’s COVID-19 registry
Source: PLoS One. 2021 Jun 3;16(6):e0252576. doi: 10.1371/journal.pone.0252576 (PMC8174679; doi:10.1371/journal.pone.0252576)
Supplement: S3 Table — (DOCX) [file pone.0252576.s003.docx]

**S3 Table. Clinical Characteristics and outcomes of hospitalized patients with COPD who tested positive for COVID-19 categorized by OCS usage.**

|  | | No OCS | OCS | p |
| --- | --- | --- | --- | --- |
| n | | 63 | 308 |  |
| Demographics | | | | |
| Male sex (%) | | 31 (49.2) | 125 (40.7) | 0.27 |
| Race (%) | |  |  | 0.176 |
| Black | | 31 (50.0) | 117 (38.4) |  |
| Other | | 1 (1.6) | 11 (3.6) |  |
| White | | 29 (46.8) | 156 (51.1) |  |
| Hispanic | | 1 (1.6) | 21 (6.9) |  |
| Age (mean (SD)) | | 68.94 (16.88) | 66.58 (13.89) | 0.238 |
| BMI (mean (SD)) | | 31.47 (10.64) | 31.62 (9.24) | 0.91 |
| Smoking status |  | |  | 0.762 |
| Current | 6 (9.8) | | 25 (8.2) |  |
| Former | 55 (90.2) | | 275 (90.2) |  |
| Never | 0 (0.0) | | 1 (0.3) |  |
| Medications (%) | | | | |
| LAMA | 5 (7.9) | | 81 (26.3) | 0.003 |
| LAMA/LABA | 2 (3.2) | | 32 (10.4) | 0.117 |
| Inhaled corticosteroid | 17 (27.0) | | 184 (59.7) | <0.001 |
| Comorbidities (%) | | | | |
| Asthma | | 9 (14.3) | 119 (38.6) | <0.001 |
| Congestive heart failure | | 22 (34.9) | 126 (40.9) | 0.457 |
| Hypertension | | 51 (81.0) | 274 (89.0) | 0.122 |
| Diabetes | | 41 (65.1) | 211 (68.5) | 0.702 |
| Obesity | | 31 (49.2) | 152 (49.4) | 1 |
| In-hospital outcomes (%) | | | | |
| Pulmonary embolism | | 7 (11.1) | 32 (10.4) | 1 |
| Sepsis | | 18 (28.6) | 84 (27.3) | 0.956 |
| Pneumonia | | 44 (69.8) | 230 (74.7) | 0.523 |
| Shock | | 13 (20.6) | 40 (13.0) | 0.167 |
| Acute kidney injury | | 31 (49.2) | 155 (50.3) | 0.981 |
| Acute liver failure | | 4 (6.3) | 12 (3.9) | 0.594 |
| DIC and coagulopathy | | 12 (19.0) | 66 (21.4) | 0.8 |
| ICU admission | | 21 (33.3) | 106 (34.4) | 0.985 |
| Endotracheal intubation | | 11 (17.5) | 61 (19.8) | 0.8 |
| Mortality | | 17 (27.0) | 54 (17.5) | 0.118 |
| Month of COVID positivity (%) | |  |  | 0.292 |
| March | | 4 (6.3) | 11 (3.7) |  |
| April | | 10 (15.9) | 29 (9.7) |  |
| May | | 10 (15.9) | 51 (17.1) |  |
| June | | 14 (22.2) | 44 (14.8) |  |
| July | | 15 (23.8) | 85 (28.5) |  |
| August | | 10 (15.9) | 77 (25.8) |  |
| Data are presented as n (%) for categorical variables and mean [SD] for continuous variables. DIC = disseminated intravascular coagulation. Month of COVID positivity represents the month during which the COVID test was positive requiring admission to the hospital. | | | | |
