# Supplementary material for: Inhaled corticosteroids do not adversely impact outcomes in COVID-19 positive patients with COPD: An analysis of Cleveland Clinic’s COVID-19 registry
Source: PLoS One. 2021 Jun 3;16(6):e0252576. doi: 10.1371/journal.pone.0252576 (PMC8174679; doi:10.1371/journal.pone.0252576)
Supplement: S4 Table — (DOCX) [file pone.0252576.s004.docx]

**S4 Table. Multivariate logistic regression analysis of COPD patients comparing those on OCS versus those not on OCS.**

|  | COPD taking OCS versus COPD not taking OCS | | |
| --- | --- | --- | --- |
|  | Unadjusted OR (95% CI) | Adjusted (model1) * OR (95% CI) | Adjusted (model 2) * OR (95% CI) |
| COVID positive | 0.92 (0.81-1.05) | 0.88 (0.77-1.01) | 0.92 (0.80-1.07) |
| Hospital admission | **1.70 (1.26-2.33)** | **1.90 (1.39-2.63)** | **1.54 (1.10-2.19)** |
| ICU admission^1^ | **1.60 (1.00-2.66)** | **1.72 (1.07-2.89)** | 1.52 (0.92-2.64) |
| Ventilator^2^ | 2.18 (0.99-5.75) | **2.30 (1.04-6.12)** | 2.10 (0.91-5.77) |
| Mortality^1^ | 0.58 (0.31-1.10) | 0.65 (0.33-1.32) | 0.69 (0.33-1.50) |

OR: Odds ratio CI: Confidence interval OCS: oral corticosteroid.

* Model 1 = Adjusted for gender race age

* Model 2 = Adjusted for gender, race, age, smoking status (current versus former), comorbidities (asthma obesity diabetes mellitus congestive heart failure hypertension), and month of COVID positivity

^1^ Cohort includes only hospitalized patients

^2^ Cohort includes only ICU patients
